# Role of Counterion in the Adsorption of Ionic Amphiphiles at Fluid Interfaces

**DOI:** 10.1021/acs.langmuir.4c01259

**Published:** 2024-12-16

**Authors:** Klaus Lunkenheimer, Katrina Geggel, Rolf Hirte, Horst Seibt, Joerg Kriwanek

**Affiliations:** † Max-Planck-Institut für Kolloid und Grenzflächenforschung, D-14476 Potsdam, Germany; ‡ Max-Planck-Institut für Kolloid und Grenzflächenforschung, D-14476 Potsdam, Germany; § Technische Hochschule Wildau, Hochschulweg 1, D-15745 Wildau, Germany; ∥ Ionys AG, Max-Planck-Str. 3, D-12489 Berlin, Germany; ⊥ ACA Berlin, Max-Planck-Str. 5, D-12489 Berlin, Germany

## Abstract

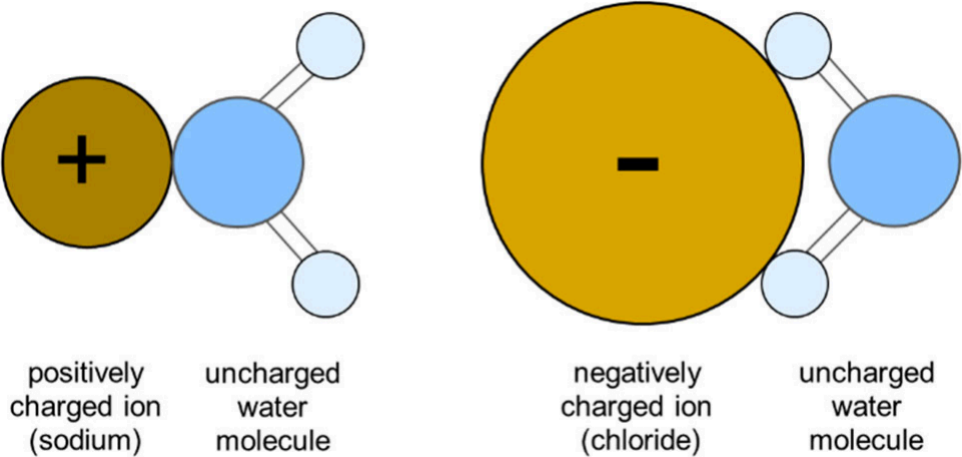

This communication represents the chemical alternative to the previous two papers dealing with the influence of positively charged alkali cations on the adsorption properties of the series of the standard surfactant system of alkali-perfluorocarbon octanoates. Now, this contribution describes the adsorption properties of the negatively charged cationic surfactant series of trimethyldodecyl-ammonium halides. In our latest contributions, we have put forward a new model of adsorption of ionic surfactants. It says that the surface excess of the adsorbed anionic surfactant is exclusively determined by the cross-sectional area of the positive counterion. This, however, has been demonstrated by applying relevant, positively charged (alkali) counterions only, i.e., by anionic surfactants. In this article, we extend the new model to negatively charged counterions (halides) applying the cationic standard surfactant series of the trimethyldodecylammonium-halides. A big difference between the hydration behavior of the positively charged alkali and the negatively charged counterions has become striking. Thus, for example, whereas the ratio between the naked ion radius of the cesium and of the lithium cation is almost 2-fold, it is practically equal for the chloride and the iodide anion. Surprisingly, however, the relevant adsorption data are practically identical. This means that the bigger, negatively charged halide counterions interact considerably more strongly with their residual ionic surfactant group than the positively charged alkali cations with theirs. Due to this, the size of the hydrated negative halide ions is considerably greater than that of the relevant positive alkali ions. These specialties can well be explained by the Stern model of charge distribution across a naked ion’s surface. It shows that for the electrostatic interaction between counterion and ionic surfactant headgroup, the peculiarities of the polar solvent of water will play a crucial role, too. By these investigations our new model of adsorption of ionic amphiphiles is further extended and gives finally evidence that it is of general validity.

## Introduction

In refs (1,2), we put forward a new model of the adsorption of ionic 1:1 amphiphiles. This was exemplified for pseudononionic conditions, i.e., under conditions of relatively high bulk concentrations when the distance between the dissolved ions is of molecular length. Due to this circumstance, solutions like these are called pseudononionic.^3−8^ These conditions were proved for the anionic standard surfactant system of alkali-perfluorooctanoates. Anionic surfactant means that its counterion carries a positive charge.

The model proposed provides experimental evidence that the cationic counterion is nonrandomly bound to its anionic amphiphilic residue. That is why the properties of the cationic counterions play a crucial role in it.

As consequence, the surface excess will be exclusively determined by the size of the hydrated counterion. Due to this, these experiments permit to additionally derive reliable information on the hydrated cations’ hydration in the boundary layer. Thus, in a way of a “scientific byproduct”, we could determine the size of the hydrated alkali counterions as exact as has so far never been possible. We then concluded that the new model is of general validity.

One reviewer of ref (2) had agreed on it, but he/she also mentioned that we need to prove it for anionic counterions, nevertheless. Right. Prosser and Franses had already criticized in 2001 that it is the bad quality of experiments that is responsible for the confusion on adsorption of ionic surfactants, but not the quality of theory.^3^ Unfortunately, this conclusion does hold yet.^3,6^ In any experimental investigation, it has to be the correctness of the experiment, which decides on the validity of any theoretical model!

However, so far, we could provide experimental evidence for the alkali cations of lithium to cesium only. Although we have already suggested in ref (2) that the novel model should be valid analogously for anionic counterions, we have been interested to provide experimental evidence for it, too. The ordinary anionic counterions are the 1:1 halide ions of chloride, bromide, and iodide.

To find out a comparable standard cationic surfactant system, we looked for one of roughly equal surface-activity, i.e., for which the pseudononionic conditions were guaranteed, too.

For it, we have available the analogous cationic amphiphile system of Trimethyl-*n*-dodecyl-ammonium halides. Their chemical formula is [(CH_3_)_3_N–C_12_H_25_)]^+^X^–^_._ The negatively charged counterions X^–^ stand for chloride Cl^–^, bromide Br^–^ and iodide I^–^ anions. Unfortunately, there are no fluoride surfactants possible.^9^ However, we found out that the pseudohalide ion of perchlorate ClO_4_^–^ may also be applied as halide anion in adsorption.

For the three analogous amphiphilic system of trimethyldodecylammonium halogenides together with the corresponding perchlorate surfactant, we have available the related equilibrium surface tension (σ_e_) versus log concentration isotherms (log c) of their surface-chemically pure solutions here.

We were interested to know whether this cationic surfactant system can prove the new model on adsorption of ionic amphiphiles put forward in refs (1,2) too.

## Experimental Section

### Purity

A stock solution of the halide surfactant was purified in an automatically operating apparatus especially developed to get surfactant solution in the required grade of surface-chemical purity (scp) as described in ref (10).

### Surface Tension Measurements

Surface tension was determined by an automatically operating LAUDA ring tensiometer taking into consideration modifications necessary to apply it to surfactant solutions.^11^ The equilibrium surface tension (σ_e_) versus log concentration (c) isotherms of the surfactants were measured by preparing dilutions of the surface chemically pure stock solution concerned. Measurements were performed at 295 K.

### Evaluation of Measurements

The σ_e_ versus log *c* isotherm can well be described by the Henry-Frumkin equation of real surface behavior,^12^ i.e., by
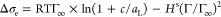
1

The symbols denote Δσ_e_ = σ_w_ – σ_e,_ surface pressure, σ_w_ surface tension of pure water, Γ_∞_ saturation adsorption (surface excess), *a*_L_ surface activity, and *H*^s^ surface interaction parameter, respecttively.

Whereas the alkali-perfluoro octanoates reveal ideal surface behavior with *H*^s^ ≅ 0, the trimethyldodecylammonium halides obey real surface behavior with a small surface interaction of 2.0 < *H*^s^ < 3.5 mN/m.

**Figure 1:**
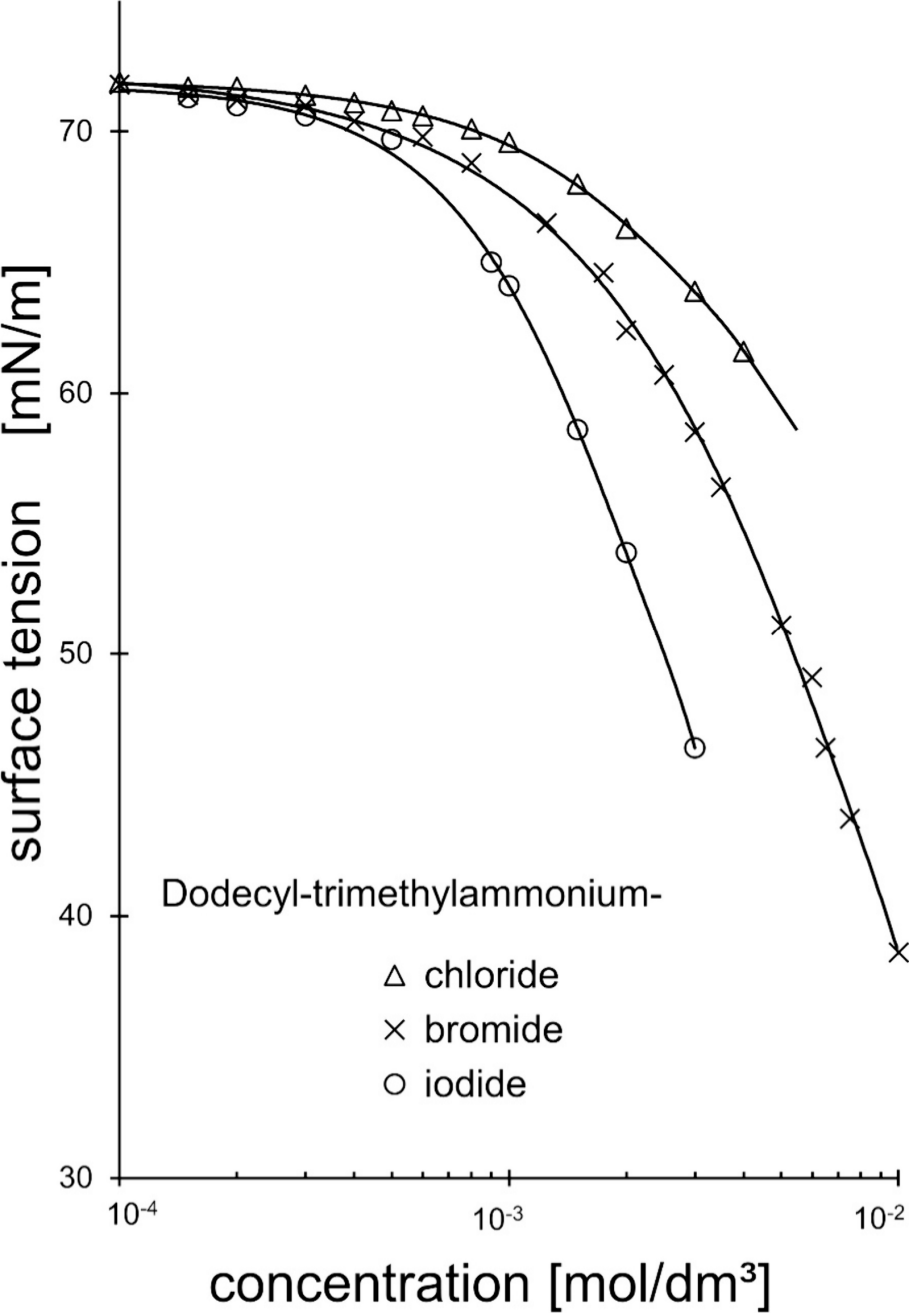
Equilibrium surface tension, σ_e_, versus log concentration isotherms of the three homologous *n*-dodecyltrimethylammonium halides of chloride, bromide, and iodide.

The σ_e_ vs log *c* isotherms of these cationic halide surfactants are plotted in Figure 1.

### Synthesis

Dodecyltrimethylammonium chloride was used “as received” (Sigma/Aldrich). Since the other two halides were not available commercially, we produced them by ourselves.

Therefore, dodecyltrimethylammonium bromide was synthesized by warming up an aqueous solution of 0.3 mol of trimethylamine (Laborchemie Apolda) together with 0.3 mol dodecyl bromide (Sigma/Aldrich). After completion of the reaction, the solution was diluted with ethanol, and the product was crystallized in a refrigerator. Thereafter, it was five times recrystallized from ethanol.^13^

Dodecyltrimethylammonium iodide was prepared by dissolving 0.2 mol of dodecyldimethylamine and 0.7 mol of methyl iodide in 30 mL ethanol. This mixture was left to stand at room temperature for 24 h. The obtained precipitate was recrystallized three times from a 1:1 mixture of dry ethyl acetate and dry ethanol.^14^

The compound *n*-dodecyltrimethylammonium perchlorate we had been presented by the Jerzy Haber Institute of Catalysis and Surface Science of the Polish Academy of Science.

As a matter of fact, the purity of the products of the cationic surfactants of the analogous series of *n*-alkyl-trimethylammonium halides is generally unsatisfactory, with respect to exact adsorption investigations. This is the more regrettable since they are applied as “standard surfactants” yet. By it, investigations dealing with adsorption properties are to be estimated critically.

Unfortunately, the isotherm of the chloride analogue is “rather short” i.e. its lowest equilibrium surface tension value amounts to 61.5 mN/m only. It turned out that this compound, which is usually used as “standard” of a cationic surfactant, was especially contaminated with some strongly surface-active substance(s). We had started the procedure of surface-chemical purification at a concentration of 4 × 10^–3^ M having a surface tension value of about 53 mN/m. However, after having performed a few hundreds of purification cycles, we could not yet reach the necessary state of surface-chemical purity (scp). Hence, we performed purification in a second apparatus likewise. We used this to dilute the first solution. This enabled us to continue purification until the required state of scp at 61.6 mN/m was reached after 1210 cycles finally. Thus, about 9 mN/m change in surface tension were only due to unknown surface-active contaminants!

**Figure 2:**
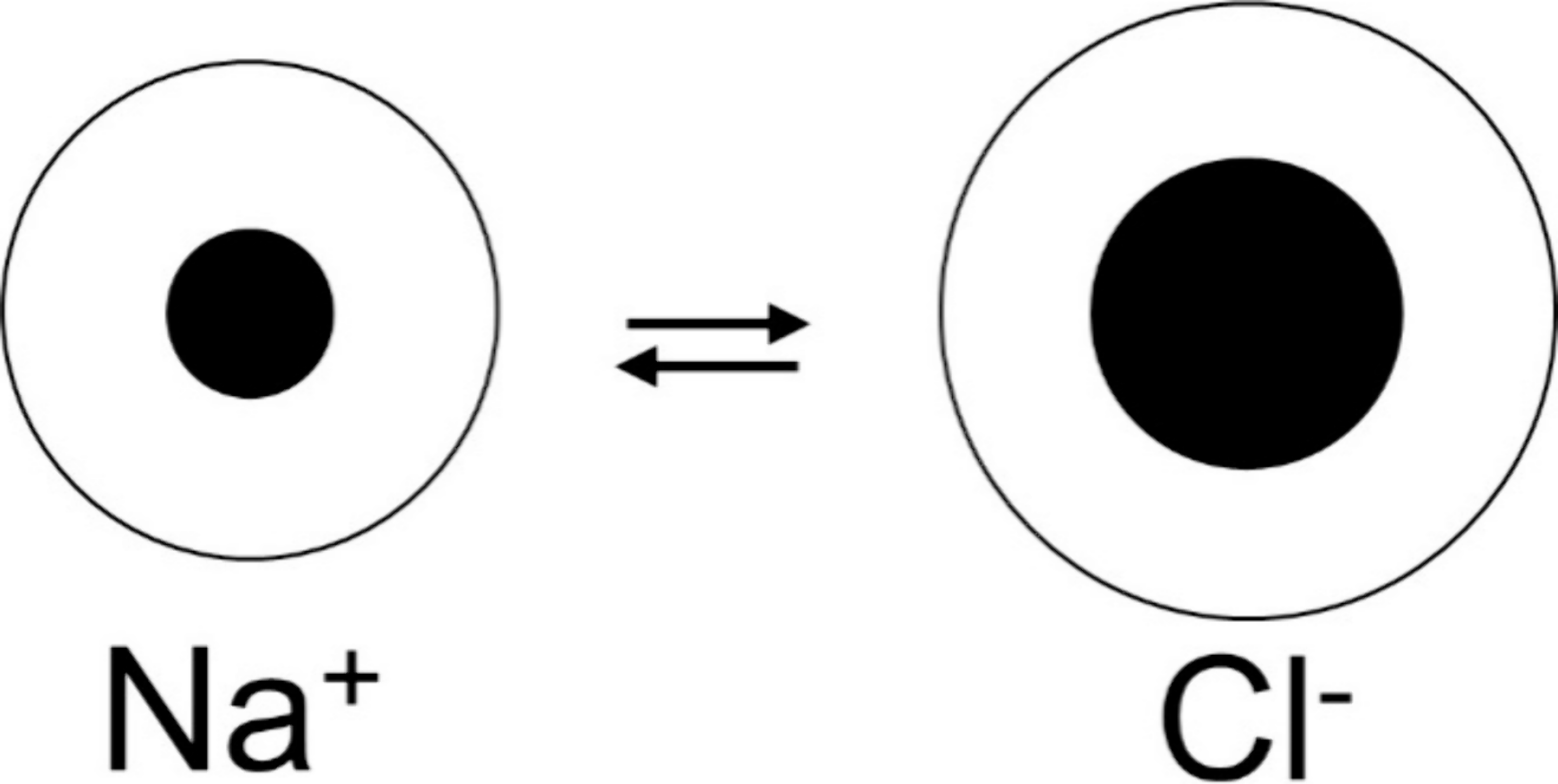
Schematic comparison of the relationship between the thickness of the hydration layer and the ionic radius for the positively charged sodium and the negatively charged chloride counterion. These two ionic counterion compounds possess almost equal hydration radii each. (Symbols roughly in scale).

**Figure 3:**
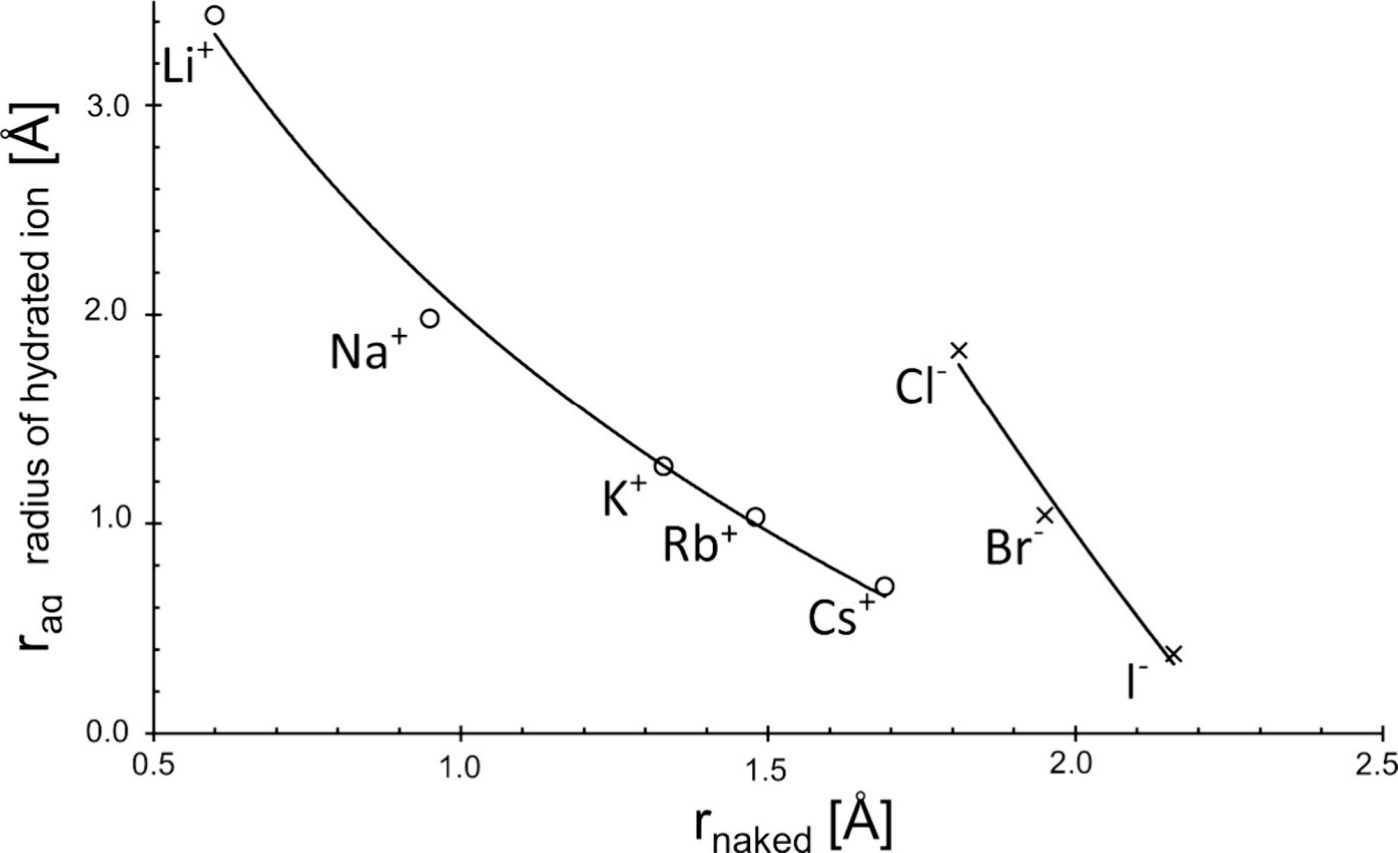
Size of hydrated positively and negatively charged counterions as a function of the radii of their related naked ions.

We decided that we preferentially should have the correct surface tension data instead of more measuring points. Figures 2 and 3 confirm that this approach was correct. The resulting effects are so big that it does not account to have a few more or less measuring points of the related σ_e_ vs log *c* isotherm.

Analogous problems were encountered with the other two surfactants. Thus, for example, the bromide compound had been recrystallized five times. However, with respect to adsorption properties, it remained very contaminated nevertheless. Obviously cationic surfactants are especially prone to contain surface-active trace impurities.^6^

These findings underline the outmost importance of surface-chemical purity anew for cationic surfactants especially. Meanwhile the importance of special purity is also observed in crystal research.^15,16^ In (17), it is written “surface-sensitive techniques depend critically on the purity grade and purification processing of the chosen salts and their solutions. This is true not only for the ACS grade salts but also for the ultrapure (UP) grade.”

Comparing the amphiphilic series of the cationic alkali counterions with those of the anionic halide counterions, you may think it to be almost analogous. There is a continuously occurring curve line of all σ_e_ vs log *c* isotherms. Steeper slopes of the isotherms go in line with greater surface excess values. The two series of cationic and anionic surfactants cover almost the whole range of surface excess for straight chain *n*-alkyl surfactants. Thus, for chloride and/or iodide it amounts to
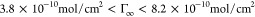
2acorresponding to cross-sectional areas per molecule adsorbed of
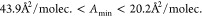
2band for lithium- and/or cesium- of the anionic alkali-perfluoro-octanoates it is
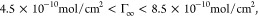
3acorresponding to
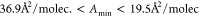
3b

Although there are only the three anionic counterions of chloride, bromide, and iodide available, they nevertheless do cover the whole range of possible surface excess. (However, as shown below, we can include the pseudohalogenide compound of the perchlorate ion, too.)

Comparing the series of anionic surfactants with that of the cationic ones, it is interesting to note that deeper insight reveals that the entire possible hydration of the positive counterions is rather different from the negative ones. This becomes evident by considering Figure 2 in which the two ions of sodium and chloride of almost equal hydration radius are drawn together including the radii of their naked ions. Whereas the positively charged counterion needs a smaller radius of the naked ion, the anionic counterion possesses a comparatively bigger naked ion radius but reaches analogous hydration layer thickness, nevertheless. This difference is more pronounced the smaller the naked ion radii are. The naked ion radius of chloride is practically twice that of sodium.

The different nature of hydration in the surrounding of a positively and/or a negatively charged counterion becomes quite evident when we plot the thickness of the hydrated ion denoted as *r*_aq_, versus its naked ions radius (Figure 3). From it you at once notice that there is a great difference in hydration between a positively and a negatively charged counterion.

Qualitatively the known trend is confirmed, saying that a bigger size of counterion will lead to smaller surface excess. However, quantitatively this trend becomes quite different with the slope of the relationship being distinctly steeper in the case of the anionic counterion.

## Results and Discussion

As mentioned in the introduction, to generalize the new model of adsorption of ionic amphiphiles, which we have successfully proved by the anionic standard amphiphile system of alkali-*n*-perfluorooctanoates, we need to validate it also for a comparable standard cationic surfactant system. This is the case for trimethyl-*n*-dodecylammonium halides.

In the literature, there is no agreement about the nature of the negatively charged counterions of the halides’ hydration. We have looked for quantitative data on the hydration layer thickness of halide ions either in the adsorption layer or in the bulk phase. Applying an Internet search by the platform Google Search for the last 50 years we did not find any quantitative experimental result, although there are numerous articles on hydration of halide anions.^18^ Thus, for example, by using the terms “trimethyldodecylammonium halide hydration”, there are about 18 thousands of publications or books dealing with practical application and theoretical calculations of this cationic surfactant in a lot of fields. By adding the term “layer thickness“, the number is reduced to around ten thousand entries. If we added the term “absolute scale”, the number would further be reduced to about three thousand entries, and if we added the term “surface-chemically-pure” the number was finally reduced to none.

Summarizing this search, the point is that the research papers usually report on convenient capabilities of this standard surfactant. They always speak of “hydrated” surfactant, in particular of hydration of its micellar surface.^5−8^ However, you will not find any quantitative data on the hydrated ions’ layer thickness in molecular scale.^18,19^ There is practically no difference whether you ask for bulk phase or adsorption layer. Even a theoretical endeavor does not help.^15^

We found one publication which streamed for answering this question.^15^ Unfortunately, however, they assumed that the surfactants’ hydration concerns the whole amphiphilic molecule. Insofar as they were right to investigate the influence of different nonionic, polar groups bound to the hydrophobic decyl chain of the trimethyldecylammonium chloride surfactant. Indeed, they were successful in finding a small effect on the surfactants’ adsorption properties. However, they did not understand that hydration of the surfactant does need a full charge, i.e., an ionic group. This is brought about exclusively by the electrostatic interaction between the totally charged counterion and the solvent molecule. In addition, by adding a polar group, you will well alter surface activity or surface enthalpy, which are bulk properties. But you will not essentially alter the amphiphilic molecule’s hydration which can favorably be followed by the surfactants’ adsorption layer properties, i.e. by their surface excess Γ_∞_. That is why they stated surprised that “the hydration of micelles formed by surfactant cations with a single alkyl chain on quaternary ammonium is approximately the same, regardless of the alkyl chain length or functionalization of the headgroup.” Of course, hydration of the pseudononionic ensemble of counterion and ionic headgroup is so strong that it will hardly be affected by substitution of the *n*-decyl chain by a group of small nonionic substituents.

**Figure 4:**
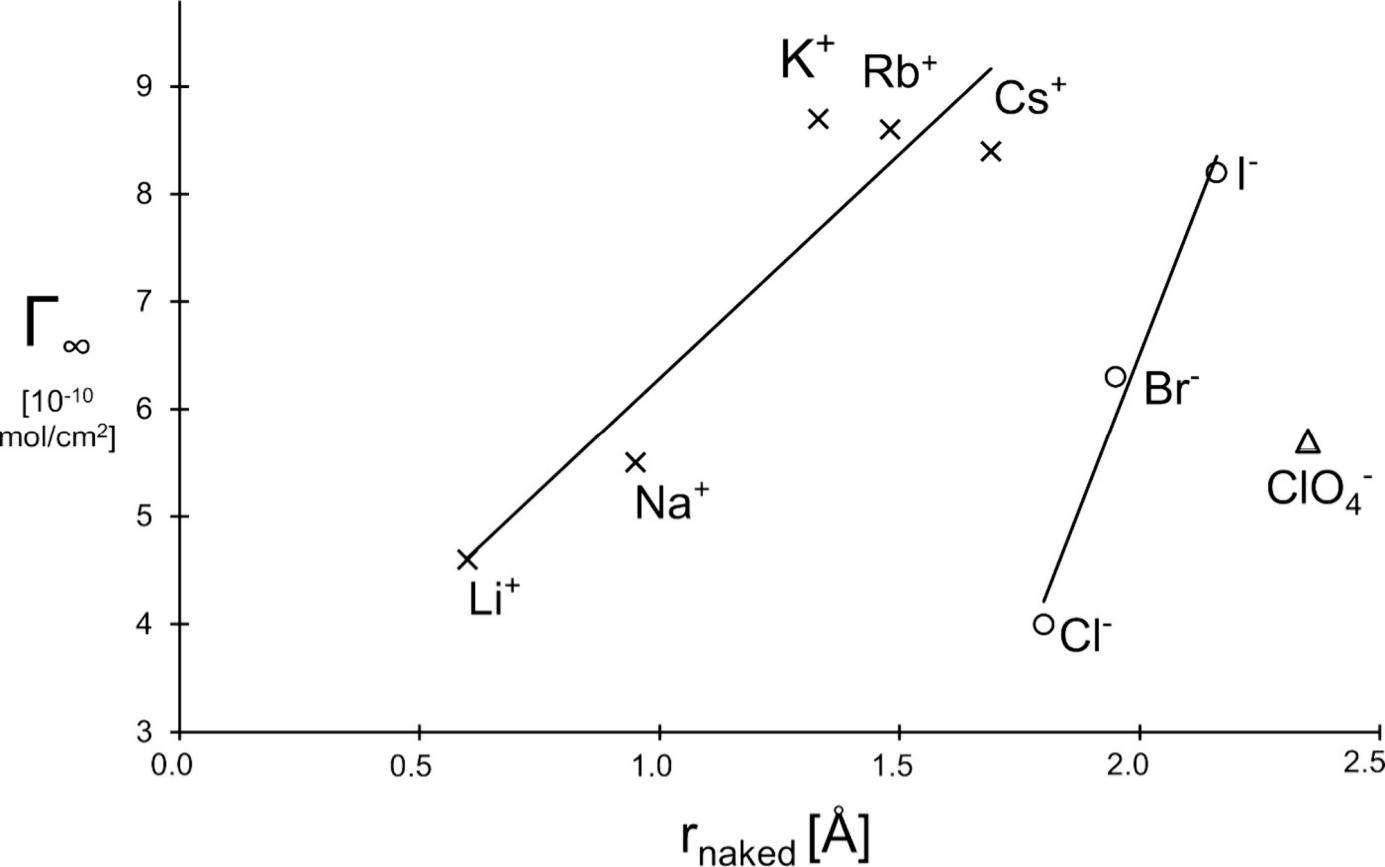
Surface excess Γ_∞_ as a function of its naked ion radius *r*_naked_.

Coming back to the outstanding question whether the hydration of a negatively charged ion does completely resemble that of a related positively charged one, we need to answer it experimentally. Interestingly, we have found now that the negatively charged counterions are even stronger hydrated than their positively charged counterparts. By it, we have reasonably to conclude that our new model of ionic surfactant adsorption does hold for the cationic surfactants analogously. To get an impression of the enormous difference, we have plotted the surface excess of these two ionic surfactant systems as a function of their naked ion’s radius (Figure 4).

This figure clearly shows the difference between surface excess of the positively and negatively charged counterions. There is also a distinct difference in the slopes of the two lines.

Realizing that the naked ion radii of chloride and of cesium are roughly equal, the surface excess of the former is approximately twice that of the latter. In other words, it means that under conditions when the heaviest alkaline ion possesses the smallest hydration layer, the lightest halide ion of chloride possesses the biggest hydration layer, about 2.6 times that of the largest alkali one. What does this mean?

Further on, there seems to be another discrepancy, i.e., that of the pseudoanion perchlorate. In (2), we just had found that the surface excess can always be explained reasonably by applying the elements’ properties valid in bulk, too. Figure 3 shows an ideal course for the three halide anions of chloride, bromide, and iodide.

**Figure 5:**
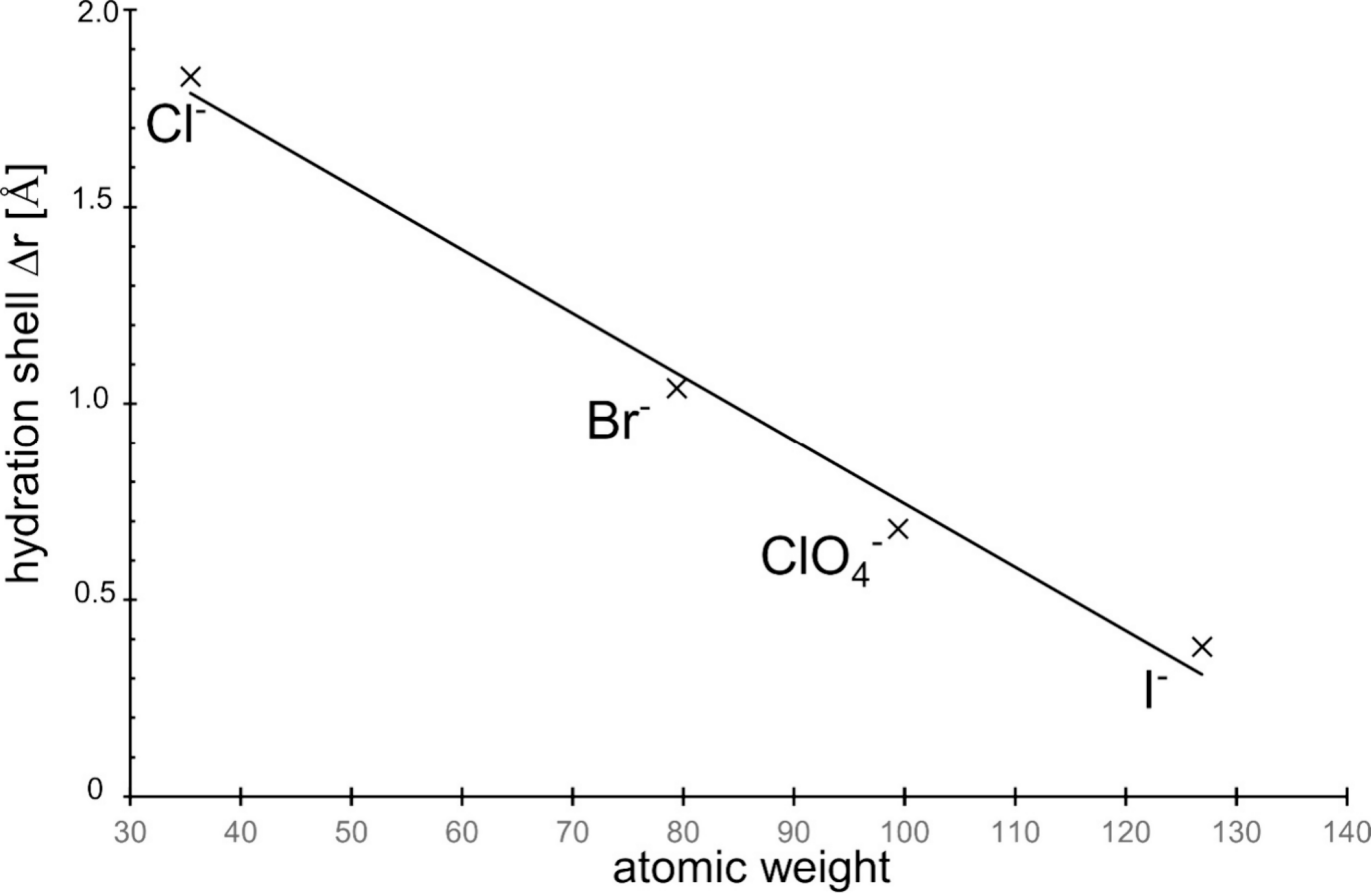
Hydration shell thickness of the halide counterions as a function of the atomic weight. The perchlorate molecule is assumed to be of pseudohalide ion nature.

Figure 4, however, makes us believe that this seemingly does not hold for the perchlorate ion, although the bulk properties of it indeed being of pseudohalide nature.^20^ Does there really exist an extraordinary exception to the general rule of chemistry? To decide whether this is true, we again remember Mendeleev.^21^ He said that the criterion to decide whether some element’s property does follow a reasonable trend is to plot this property as a function of the atomic weight. However, perchlorate itself does not represent an ion of an element but rather an ion of a molecule. But, treating this molecule as a “pseudo-element”, we arrive at Figure 5. In it, the thickness of the ions’ hydration layer Δ*r* measured by adsorption is plotted as a function of the atomic weight. Doing so, we obtain an almost ideal relationship within limits of error. The correct value of it is Δ*r*^+^ = 1.04 Å then. Thus, the perchlorate anion can well be treated as a pseudohalide one, even in adsorption properties.^16^ Just a “byproduct” result appears!

The seemingly exception of the perchlorate anion in Figures 3 and 4 is obviously due to a too great crystal ion radius of the chlorate ion being 2.36 Å, taken from the table of ref (22). In ref (23), the perchlorate and the iodide ion are compared in their interaction with the water molecule. Therein the radii of both ions are practically equal. In ref (24), the perchlorate ion’s radius obtained from diffusion measurements is reported to be much smaller, namely 1.37 Å. If we plot the ionic radii of chloride, bromide, and iodide versus their atomic weight and interpolate from this ideal straight line at the molecular weight of perchlorate (M = 100) the related “ideal” value for its naked ionic radius a value of 2.02 Å is received. The latter value is well in line with the halide ions’ physicochemical behavior, where perchlorate characteristics lay always between the ones of bromide and iodide. Thus, an ionic value of 2.36 Å for the perchlorate ion taken from ref (22) seems to be about 10% too great.

Now, to understand the enormous differences between the positively and the negatively charged counterions, we need a reasonable reference state. To do so we assume that the interaction of the water dipole with the charged surface is mainly determined by electrostatic interaction.^22−29^ This is mostly assumed and was already assumed by Langmuir to be valid.^30,31^

Searching for the reference state we reread Otto Stern.^32^ He treated a simple ion’s surface charge distribution across its surface, supposing the ion to be spherical. He assumed that all univalent ions carry an equal unit of charge, whether it be positive or negative. However, it is the ions’ size that is different.

Stern then defined the charge per area as “potential”. To avoid confusion with the experimentally measurable “surface potential”^33^ we call it “surface charge”.

Thus, it is the charge distribution across the ion’s surface that must vary. To apply the charge distribution across the whole surface, we calculate that percentage of charge which is “accommodated” in a unit surface compartment of one square Ångstrom. We call it the relative surface charge *q*. By it, we can discriminate the electric properties of each ion individually.

We now assume that the hydration is related to the ions’ electric properties.

**Figure 6:**
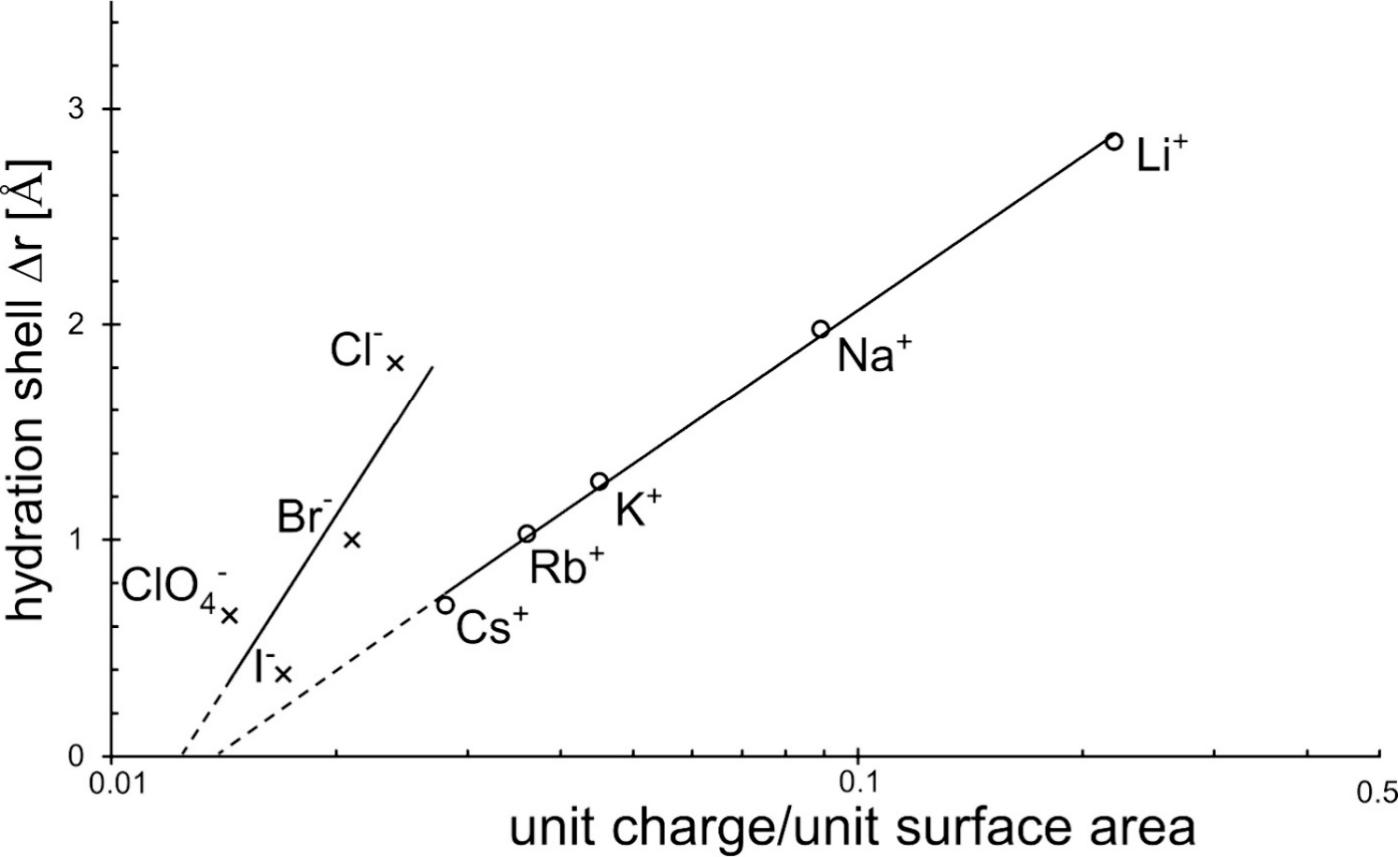
Thickness of the hydration shell as a function of the relative surface charge of the counterion.

In Figure 6 we plotted the hydration layer thickness Δ*r*_aq_ as a function of the relative surface charge q.

In the following, we shall omit the indices. Thus, Δ*r*_aq_ is calculated by the difference between the size of the hydrated counterion obtained from the adsorption data (*r*_aq_) and the related naked ion’s radius obtained from crystal data (*r*_ion_)
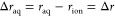
4

To get a reasonable reference point valid for positively and negatively charged counterions, we define a “standard value *q*_0_” as
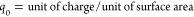
5a

Quantitatively this is
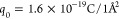
5b

To receive the relative ion’s surface charge *q*, we simply divide the “standard surface charge” by the ion’s surface area, i.e.
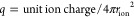
5c

Treating the thickness of the ions’ hydration layer as a function of its surface charge *q* leads to Figure 6.

This figure represents a lot of interesting information.

First, the homogeneous course of the trend detectable already in Figure 4 is confirmed, even though it is more accurate. However, the trend is inverse, i.e. the same hydration is received by ions of lower relative surface charge.

Second, the two curves are evidence of the correctness of the measured experimental data, surfactant adsorption, and X-ray analysis. Hence it is another hint on the experimental requirement to apply adsorption layers of sufficient purity in the case of correct investigation.

Third, we find that the electrostatic interaction of the negative halide ions with the solvent water is much stronger than with the positive alkaline cations, although the kind of interaction seems to be alike, i.e. higher surface charge density goes in line with higher hydration layer thickness. Taking the two ions of chloride and of sodium having almost equal hydration radii of roughly 2 Å, we see that the chloride ion needs only a relative surface charge as low as 0.024, but the analogous sodium ion needs almost four times of it to behave alike.

These experimental examples clearly illustrate the negatively charged counterions’ much stronger electrostatic surface interaction with the solvent water.

Fourth, although we observe such great difference, we also note that the two different lines of positive and negative counterions do meet at practically identical relative surface charge density in case the hydration layer thickness becomes zero, related to a naked ion radius of 2.30 Å. This means that at a surface charge lower than it there will never be possible any electrostatic interaction with the polar solvent of water, and may the counterion be positively or negatively charged. By it, we are enabled to estimate the limit of possible ion hydration quasi as another “scientific byproduct”.

To understand these features, we again refer to the scientists of the old school.^30−32^ They tell us that simple counterions will always carry the same uniform surface charge density except for their electric sign, as long as they are of identical geometrical size. This spherical case is practically encountered for the ions of cesium and of chloride. As we have found now, the thickness of the chloride ion’s hydration layer is almost 3-fold that of the cesium ion one’s. What is the enormous difference in their hydration behavior due to (cf. Figure 3)?

If you apply equal relative surface charge, you ought to expect equal sizes of hydration for both kinds of counterion, being of negative or positive charge, provided all conditions of interaction remain unaltered.

As we can take from Figure 6 equal relative surface charge is practically given for the ions of chloride and of cesium. However, under these conditions hydration of chloride is already maximal then, i.e., two and a half that of cesium. Therefore, we can either assume some extra force acting, or we have to assume that the solvent around the two oppositely charged ions will also be involved somehow.

On this background, we remember the properties of the solvent, i.e., of the water molecule. The water molecule itself, although being neutral, does not have equal charge distribution across its size. On the contrary, the water molecule does form a dipole with discontinuous distribution of charge across its configuration.

**Figure 7:**
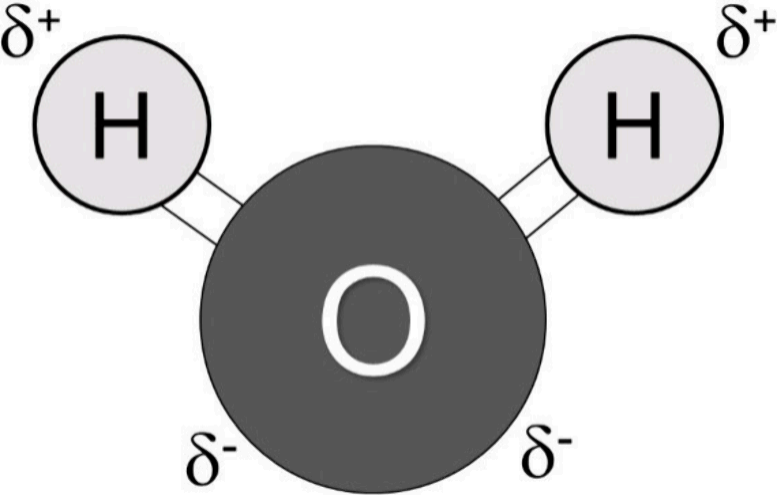
Symbolic drawing of the charge distribution across the dipole of water. The letters of δ symbolize the discontinuity of charge across the dipole.

On principle the water molecule represents a quadrupole consisting of two OH-dipoles.^34−39^ As the oxygen nucleus attracts electrons more strongly than does the hydrogen nucleus, oxygen is more electronegative^37,38^ (Figure 7). We represent a few examples of the many pictures of the Google Search in References.^34−40^

The sharing of electrons between H and O is therefore unequal. This unequal electron sharing creates two electric dipoles in the water molecule, one along each of the H–O bonds. The oxygen atom bears a partial negative charge (2δ−), and each hydrogen a partial positive charge (δ+).^26,27,37,38^

The bond length between the hydrogen and the oxygen atom amounts to almost 1 Å (0.96 Å). The obtuse angle between the three atoms of hydrogen and oxygen is 104.5 degrees.^18^

Researching the literature with the terms “water molecule quadrupole” you will find almost ten million references (!). Furthermore, there are several hundreds of different pictures of the water molecule.^18^ However, although numerous theoretical calculations on the accurate structure of water have been undertaken, neither of the applied theoretical models is entirely satisfactory.^34−36^ In a recent work, the situation on “ion specific effects” in surfactant adsorption layers is cited as the following “Since then (Poiseuille 1884) “ion specificity” has been discussed at great length for over 100 years, yet there is no generally accepted first-principles theory of it.”.^37^

**Figure 8:**
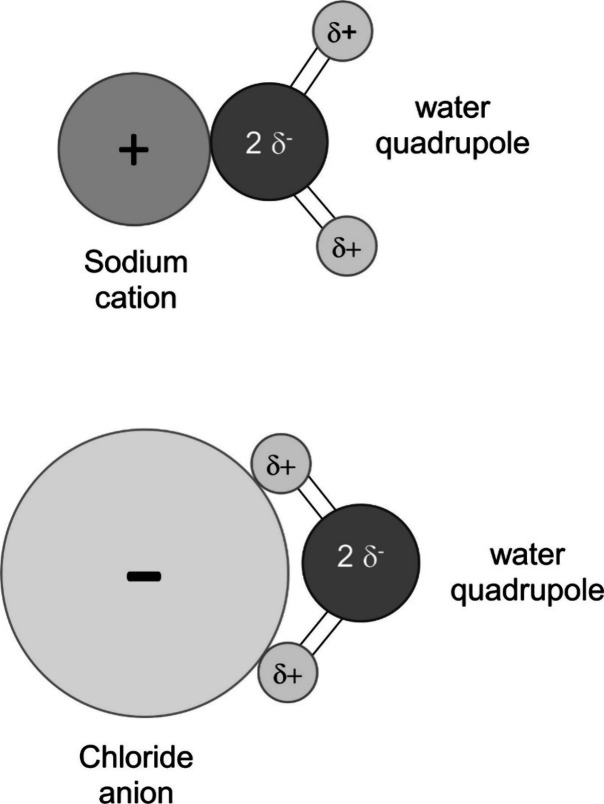
Symbolic representation of the water dipole’s orientation within the hydration layer of either negative or positive counterion. The sodium and the chloride ion possess almost equal hydration layer thickness but different radii of their naked ions. Darker color of ions stands for higher relative surface charge (drawing roughly in scale).

Now applying the charge distribution across the atomic surface on the dipole of the water, it is reasonable to assume that the water molecule will arrange around the counterions’ surface as shown in Figure 8.

We have chosen the two ions of hydrated sodium and/or chloride that are of almost equal size. The water molecule is also practically in size. If we refer to electrostatic interaction, the water molecule’s orientation in the hydration layer should be in such way that the ion possesses opposite sign of charge. From this figure, it becomes obvious that the water molecules’ orientation will be different in both alternative states. Thus, we can at least qualitatively understand why the hydration around the positive counterion is different from that around the negative one. Of course, we cannot conclude in which case the interaction will be stronger. It is important to emphasize that this information is only received from experimental findings. However, we did not imagine that the discrepancy will be so strong like it.

**Figure 9:**
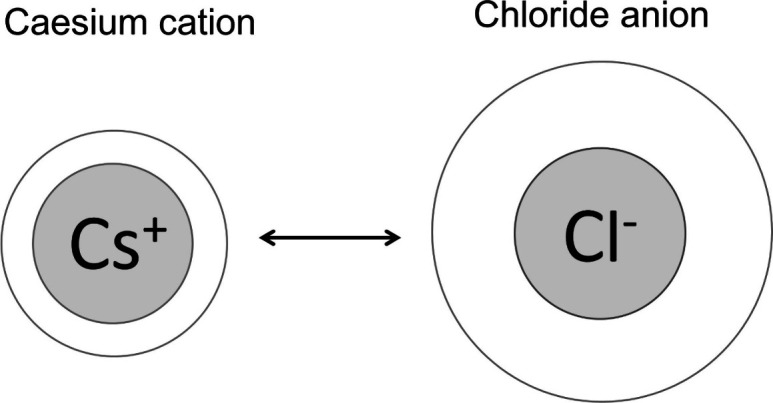
Hydration layer thickness of chloride anion and sodium cation. These ions have almost equal naked ion sizes but quite different hydration layer thicknesses (empty space).

The different hydration layer thickness of the two ions of chloride (anionic) and cesium (cationic) becomes visually quite evident in Figure 9 for which the two naked ions have almost equal size. The dimensions are given roughly in scale.

Considering the size of the trimethylammonium group, we remember that the covalent distances of the atoms are as allows: C–H = 1.10 Å, C–N = 1.47 Å.^57,58^ In ref (58), it is shown that the size of the cationic group is not greater than that of typical anionic surfactants, like those of alkylsulfates and of alkansulfonates.

Using these experimentally well-established results may be a convenient impetus for the theoreticians to get deeper insight into this problem.

At least, we hope that our investigations will help to provide these molecular properties, which are really required to describe surfactant adsorption satisfactorily, avoiding those molecular characteristics which are of less importance. There are too many of the latter under investigations.^54−56^

## Conclusions

1. The investigations on the adsorption of the cationic standard surfactants of trimethyl-*n*-dodecyl-ammonium halides fully confirm the new model on the adsorption of ionic amphiphiles put forward by us recently in refs (1,2). It says that for extended chain surfactants, it is only the size of the counterion that determines the surface excess, because the hydrated counterion has the bigger diameter and is located always directly to (below) the surfactant’s headgroup.
2. The hydration shell of a counterion is of well-defined size. Its thickness is an inverse function of the diameter of the naked ion’s size, irrespective of whether the counterion’s charge being positive or negative.
3. Opposite to the nature of the water molecules in the bulk, where they are randomly distributed, they will have a nonrandom arrangement in the hydration layer. Thus, the physicochemical properties of the hydrated water molecules will be somehow different from them in bulk.
4. In addition, it turned out that the characteristic of hydration depends on the sign of the counterion. Negatively charged counterions, such as halides, do stronger interact with water molecules than positive ones, such as alkali ions. Thus, there will obviously be another arrangement of the former somewhat different from that of the latter.
  To understand it reasonably, we have to realize that the polar water molecule itself represents a dipole which will have different orientation around the ionic counterion as a function of its charge. From chemical point of view this is to be expected. From Figure 8, it intuitively becomes evident that the water molecule’s interaction with the negatively charged ion will be stronger. This difference is enormous. The different hydration layer thickness of the two counterions of chloride (anionic) and of sodium (cationic) becomes quite evident visually in Figure 9, in which the two naked ions have almost equal size. The dimensions are given roughly in scale.
5. By plotting the hydration layer thickness Δ*r* versus the unit of charge the big difference in hydration around a positively or a negatively charged counterion becomes striking. The electrostatic interaction between the polar water molecule and the ionic counterion becomes distinctly stronger for the negatively charged anions.
  In addition, it is instructive that the two different curves of Δr vs relative surface charge originate exactly at the same value of ion radius in case hydration becomes negligible. It means, on one hand, that the kind of interaction is the same in both cases, namely electrostatic in nature.^29^ On the other hand, it says exactly how long this interaction will reach, i.e. its radius extends maximal to about 2.2 Å. Thus, it gives us that size of a naked ion which will no longer get hydrated any more.
6. This, in turn, would mean that the physicochemical properties of water molecules in the hydration sphere of counter-anions should also be somewhat different from the ones present in hydration sphere of the counter-cations. It means that the structure of water within the ions’ hydration layer will not only depend on the counterions’ charge density but also on its sign of charge.
  Thus, there will also be different properties of the water molecule possible. Concerning hydration there are at least three different ones, i.e. those of the conformation of water molecule within the bulk phase and/or within the hydration layer around a positive and/or a negative counterion, respectively.
7. These results reveal that you must not blame the theoreticians dealing with molecules and ions for establishing false models of ionic surfactant adsorption.^41,42^ It is rather the insufficient and/or false experiments on ionic adsorption that has led theoreticians to elaborate false models. This has already been stressed by Prosser and Franses^3^ and by Kohler and Semmler^16^ over 20 years ago.
8. Concerning correctness of experiments, we come to an important conclusion. In investigating the ionic properties of adsorbed amphiphiles, it is of the utmost importance to apply a convenient experimental method which is favorable to follow alteration in the adsorption layer. In many cases, there are applied complicated methods which are favorable in monitoring bulk rather than monolayer properties.^2^
  Surface tension represents itself a measuring method which stems practically exclusively from adsorption layer properties. Being related to the boundary layer of the liquid surface means being necessarily related to the surfactant’s concentration in the boundary layer. If you recalculate the boundary layer concentration in terms of bulk concentration by relating it to a thickness of ten or one hundred of Angstrom leads to a concentration of one or ten mol/L. This circumstance is the main point that surface tension measurement is most suitable in following every tiny alteration in the adsorption layer. You have available seven hundred digits of the measuring quantity of surface tension with a measuring accuracy of it ∓0.1 mN/m that does arise exclusively from the adsorption layer. This also means that the amphiphile’s three-dimensional concentration in the boundary is usually higher by 1 to 3 orders of magnitude than in its adjacent bulk phase.
  Thus, in case there is applied an investigating method that needs such high bulk concentration to get a significant measuring signal it is suspicious as long as the measuring signal does arise mainly from the bulk properties instead from the boundary.
9. Our latest publications on adsorption of ionic surfactants underlined the new finding that the surfactants surface excess is exclusively determined by the size of the counterion.^1,2^ However, we did derive this for the positive alkali counterions only. We are now able to show that negative counterions will behave simultaneously, even to a stronger extent. Thus, the size of the hydrated halide ions’ diameter becomes exactly accessible.
  This result, however, must not mislead us to think that it is of general validity. So far, we have been dealing with dynamic and equilibrium surface tension of pseudononionic surfactants when the counterions are fixed so close to the surfactants’ ionic headgroup that the two oppositely charged ionic groups can be considered as one single nonionic polar group. However, we would like to admit the option that the size of the amphiphiles headgroup may be bigger than that of its adjacent counterion. In this case it should be the size of the ionic headgroup which determines the surface excess, but not that of the counterion.
10. We again stress, that these results were only received since we were able to guarantee the required grade of purity, which we have called “surface-chemical purity” some decades ago.^10,43−45^ The related apparatus needs to be addressed always. Interestingly there are other disciplines which found that the substances’ purity is of utmost importance, too.^10,41,42,46,47,51^
11. These facts make us conclude that there will be deeper insight into the true nature of ionic surfactant adsorption as soon as theory will take up these new findings. There is no need to apply strange assumptions on special molecular properties in the boundary layer, such as penetration of counterion, different spheres of hydration layers around the same ion, different immersion depth of the hydrophobic chain, quite different structure of ionic surfactants’ boundary with a diffusion layer, and/or some extra forces, etc.^1,2^ It is enough to apply the molecular properties valid in the bulk phase. Looking for more complicated systems, such as those dealing with divalent cations like magnesium or calcium, should fundamentally be no problem.^1,2,20,22,30,31,48,52,53^ We do not require peculiar properties of the different ions. We only need the ions’ relevant relative charge density as well as the ions’ sign.
  Maybe by following these predictions there will occur a real breakthrough in understanding the nature of ionic surfactant adsorption, which represents still a burning problem.^49,50^
